# Analysis of Therapeutic Effect of *Ilex hainanensis* Merr. Extract on Nonalcoholic Fatty Liver Disease through Urine Metabolite Profiling by Ultraperformance Liquid Chromatography/Quadrupole Time of Flight Mass Spectrometry

**DOI:** 10.1155/2013/451975

**Published:** 2013-08-27

**Authors:** Jing-jing Li, Jie Yang, Wei-xi Cui, Xiao-qing Chen, Gang-ling Chen, Xiao-dong Wen, Qiang Wang

**Affiliations:** ^1^State Key Laboratory of Natural Medicines, China Pharmaceutical University, Nanjing 210009, China; ^2^School of Traditional Chinese Medicine, Capital Medical University, Beijing 100069, China

## Abstract

Nonalcoholic fatty liver disease (NAFLD), the most common form of chronic liver disease, is increased worldwide in parallel with the obesity epidemic. Our previous studies have showed that the extract of *I. hainanensis* (EIH) can prevent NAFLD in rat fed with high-fat diet. In this work, we aimed to find biomarkers of NAFLD and investigate the therapeutic effects of EIH. NAFLD model was induced in male Sprague-Dawley rats by high-fat diet. The NAFLD rats were administered EIH orally (250 mg/kg) for two weeks. After the experimental period, samples of 24 h urine were collected and analyzed by ultraperformance liquid chromatography/quadrupole time of flight mass spectrometry (UPLC-Q-TOF). Orthogonal partial least squares analysis (OPLSs) models were built to find biomarkers of NAFLD and investigate the therapeutic effects of EIH. 22 metabolites, which are distributed in several metabolic pathways, were identified as potential biomarkers of NAFLD. Taking these biomarkers as screening indexes, EIH could reverse the pathological process of NAFLD through regulating the disturbed pathway of metabolism. The metabolomic results not only supply a systematic view of the development and progression of NAFLD but also provide a theoretical basis for the prevention or treatment of NAFLD.

## 1. Introduction

Nonalcoholic fatty liver disease (NAFLD), the most common form of chronic liver disease, is increased worldwide in parallel with the obesity epidemic. It ranges from steatosis to nonalcoholic steatohepatitis (NASH), with or without fibrosis and cirrhosis [1]. Nowadays, the most reliable methods for NAFLD diagnosis include imaging techniques such as ultrasound and magnetic resonance imaging and liver biopsy [2]. However, imaging techniques are expensive and nonspecific while liver biopsy is an invasive and subjective procedure, associated with potential complications and prone to sampling error [3]. Therefore, there is an urgent need to discover novel biomarkers to allow the reliable, noninvasive diagnosis of NAFLD. On the other hand, in spite of the advance in drug treatment, there is currently no generally accepted medical therapy for NAFLD [4]. Thus, it is essential to search for high-effective agents that would ameliorate NAFLD.

Numerous studies have suggested that traditional Chinese medicine (TCM), because of their special characteristics such as multi-ingredient, multitarget, and low adverse effects, can be important modulators in the prevention of a variety of chronic diseases [5, 6]. *Ilex hainanensis* Merr., distributed mainly in the southern region of China, is used in traditional Chinese medicine as an antihypertensive, antilipemic, cholesterol-lowering, and anti-inflammatory agent [7]. Its leaves are used as a traditional tea product, known as Shan-Lv-Cha. Patients with NAFLD treat themselves by boiling its leaves in water and drink the extract as a folk medicine [8]. Our previous studies also showed that the extract of *I. hainanensis* (EIH) can prevent NAFLD in rat fed with high-fat diet [9]. Though many phytochemical and pharmacological researches [9–11] have been carried out to study the effect of EIH on NAFLD, it remains a difficult task to clarify its mechanism of the pharmacological action due to the complexity of active compounds and the unknown synergistic actions of multiple components.

Metabonomics, offering a physiologically holistic, noninvasive platform, has shown great potential in understanding disease mechanisms and identifying diagnostic biomarkers or drug targets [12, 13]. Recent technological breakthroughs have provided researchers with the capacity to measure hundreds or even thousands of small-molecule metabolites in a few minutes, paving the way for the identification of novel biomarkers in diagnostic pathology [14, 15]. The metabolomics has aided in the development of diagnostics and therapeutics in a number of clinical areas [16, 17]. Meanwhile, it constructs a unique “fingerprint” through monitoring entire pattern of low molecular weight compounds in body fluids rather than focusing on individual metabolites, reflecting the terminal symptoms of metabolic network of biological systems in holistic context [18, 19]. This trait is well coincident with the integrity and systemic feature of TCM, indicating it is a potential tool for us to study the underlying efficacies and therapeutic mechanisms of TCM.

In this work, to find biomarkers of NAFLD and investigate the therapeutic effects of EIH, a UPLC-Q-TOF method based on a metabolomic strategy was applied to generate metabolite profiles of urine collected from normal, NAFLD model, and EIH-treated rats.

## 2. Materials and Methods

### 2.1. Chemicals and Reagents

Acetonitrile and formic acid (HPLC grade) were obtained from ROE Scientific Inc. (Newark, USA) Methanol (HPLC grade) was purchased from Jiangsu Hanbon Sci. & Tech. Co. Ltd. (Nanjing, China). Purified water prepared by the Millipore system (Millipore, Bedford, MA, USA) was used for all the preparations. Other reagents were of analytical grade. Citrate, glycine, hypoxanthine, L-cysteine, L-tryptophan, and nicotinic acid were obtained from Solarbio (Shanghai, China). Allantoin was purchased from FeiYu Biological Technology (Nantong, China).

The kits for measurement of triglyceride (TG), total cholesterol (TC), high-density lipoprotein cholesterol (HDL-c), low-density lipoprotein cholesterol (LDL-c), alanine transaminase (ALT), aspartate aminotransferase (AST), and fasting blood glucose (FBG) were purchased from Jiancheng Bioengineering Institute (Nanjing, China).

### 2.2. Plant Material and Preparation of EIH

The leaves of *Ilex hainanensis* Merr. were collected in June 2008 from a natural habitat in Guangxi, China, and authenticated by Professor Qiang Wang, Department of Chinese Materia Medica Analysis, China Pharmaceutical University, Nanjing, China where a voucher specimen has been deposited (no. 20080202).

After proper cleaning, the *I. hainanensis *leaves were extracted with 80% ethanol under reflux twice for 2 hours each. The extract was dried with vacuum distillation to yield the extract of *I. hainanensis* (EIH, yield 16.6% w/w with respect to dry crude drug). During the experiment, an appropriate amount of EIH was suspended in 0.5% sodium carboxymethyl cellulose (CMC-Na) before administration to the animals.

**Figure 1:**
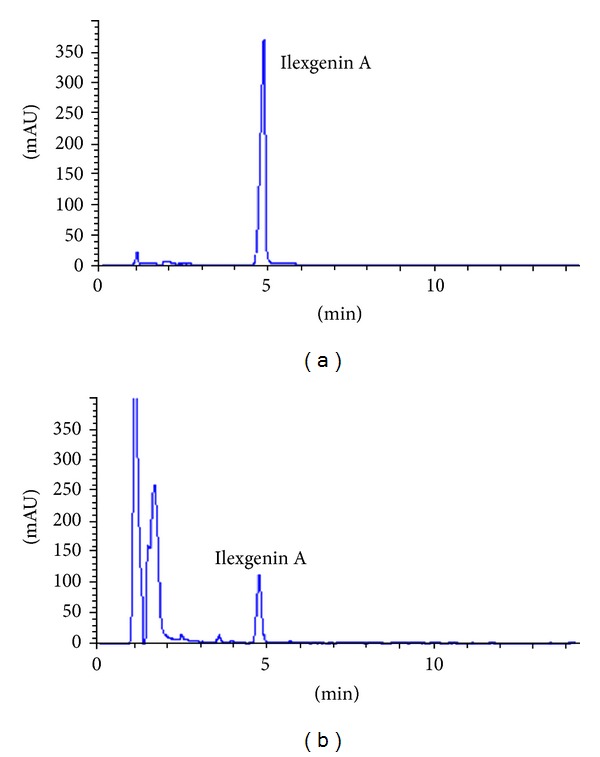
HPLC-UV (210 nm) chromatograms of the standard compound of Ilexgenin A (a) and a sample extract of *I. hainanensis *(b).

Ilexgenin A is one of the main constituents in EIH. The determination of ilexgenin A in EIH was performed on an Agilent series 1100 HPLC instrument (Agilent, Palo Alto, CA, USA), consisting of a quaternary pump, a diode-array detector (DAD), an autosampler, a column compartment, and a C18 column (Kromasil, 4.6 × 150 mm). The mobile phase consisted of 0.1% (v/v) formic acid and acetonitrile at the ratio 35 : 65 with a flow rate of 1 mL/min. The DAD was set at 210 nm. The column temperature is set in room temperature. The chromatograph has been shown in Figure 1. The percentage of Ilexgenin A was (2.01 ± 0.17)%.

### 2.3. Animals

Normal male Sprague-Dawley (SD) rats (SPF) weighing 120–150 g were purchased from Zhejiang Experimental Animal Center. Five rats were placed in one cage and maintained under controlled room temperature (20 ± 2°C) and humidity (60–70%) with day/night cycle (12 h/12 h). All animals had free access to food and water. The animal care and study protocols were maintained in accordance with the Provisions and General Recommendation of Chinese Experimental Animals Administration Legislation.

### 2.4. Animal Handling and Sample Collecting

After acclimatization for 5 days, all rats were divided into normal group (*n* = 12) and NAFLD group (*n* = 19) randomly. Normal group was given normal diet and NAFLD group was given high-fat diet, in which 10% lard (w/w), 3% sucrose, 1% cholesterol and 0.2% sodium cholate were added into normal diet [20]. After 4 weeks, all rats were fasted overnight for 12 h, and retro-orbital bleeding was conducted for analysis of TG, TC, LDL-c, HDL-c, ALT, and AST in plasma. Besides, five rats in each group were sacrificed randomly, the livers were collected, and pathological changes in the liver tissues were observed by H&E staining. 

After the success of making the NAFLD model, the rats in NAFLD group were randomized into NAFLD group (*n* = 7) and EIH-treated group (*n* = 7). Both of the two groups were given high-fat diet and normal group (*n* = 7) was given normal diet as before. In addition to the diet, the EIH-treated group was administered EIH orally once a day at a dose of 250 mg/kg for 2 weeks, while 0.5% CMC-Na was given daily in normal group and NAFLD group. After the experimental period, samples of 24 h urine were collected and stored in tubes containing 0.1 mL sodium azide solution (1% w/v) and frozen immediately at −80°C until use. At the end of experiment, retro-orbital bleeding was again conducted after overnight fasting for testing the plasma level of TG, TC, LDL-c, HDL-c, ALT, AST, and FBG. In addition, all rats were sacrificed and liver samples were weighted and collected for histopathology assessment by H&E staining. Body weight of the rats was taken on a weekly basis.

### 2.5. Sample Preparation

For UPLC-MS analysis, 300 **μ**L of methanol was added to 100 **μ**L aliquots of urine. The mixture was vortex-mixed vigorously for 2 min and subsequently centrifuged at 14,000 g for 10 min at 4°C. The supernatant was transferred to autosampler vial kept and an aliquot of 5 **μ**L was injected for UPLC-MS analysis.

To examine the stability of LC-MS system, 20 **μ**L from each urine sample was pooled to generate a pooled quality control (QC) sample and aliquots of 100 **μ**L of this pooled sample were extracted by the same method. Moreover, a random urine sample was divided into six parts and extracted by the same method. These six samples were continuously injected to validate the repeatability of the sample preparation method.

### 2.6. UPLC-Q-TOF-MS Conditions

The UPLC-MS analysis was performed on an Acquity UPLC system (Waters, Milford, MA, USA) coupled with a Micromass Q-TOF premier (Waters MS technologies, Manchester, UK). Chromatographic separations were performed on an Acquity BEH UPLC C_18_ column (100 mm × 2.1 mm, 1.7 *μ*m, Waters, Milford, MA, USA) maintained at 40°C. The mobile phase consisted of 0.1% formic acid (A) and ACN modified with 0.1% formic acid (B). The following gradient program was used: 1%–20% B at 0–3 min, 20%–60% B at 3–5 min, 60%–100% B at 5–12 min, 100% B at 12–14.5 min, and 100%–1% B at 14.5–18 min, followed by reequilibrated step of 3 min. The flow rate was 0.4 mL/min and the injection volume was 5 **μ**L.

An electrospray ionization source (ESI) interface was used and set in both positive and negative modes. The following parameters were employed: desolvation gas, 600 L/h; cone gas, 50 L/h; desolvation temperature, 300°C; source temperature, 100°C; capillary voltage, 3000 V; sampling cone, 35 kV; extraction cone, 4 V for positive mode; desolvation gas, 700 L/h; cone gas, 50 L/h; desolvation temperature, 350°C; source temperature, 100°C; capillary voltage, 3000 V; sampling cone, 50 kV; extraction cone, 4 V for negative mode. Collision energy was set at 35 eV in MS/MS mode for identification of potential biomarkers. Lock spray was utilized to calibrate accuracy of mass. Leucine enkephalin was used as the lock mass (*m/z* 556.2771 in the positive mode and 554.2615 in the negative mode). The mass range was set at *m/z* 50–1000. 

### 2.7. Data Processing

The LC-MS data were exported by Micromass MarkerLynx applications manager version 4.1 software (Waters Corporation, Milford, MA, USA). Before multivariate analysis, the data of each sample was normalized to the total ion intensity per chromatogram. The orthogonal partial least square (OPLS) and principle component analyses (PCA) were performed by the SIMCA-P 11 version (Umetrics AB, Umea, Sweden). The significance was expressed by using Student's *t*-test. *P* values less than 0.05 were considered significant. 

## 3. Results and Discussion

### 3.1. EIH Improved NAFLD Induced by High-Fat Diets in Rats

**Figure 2:**
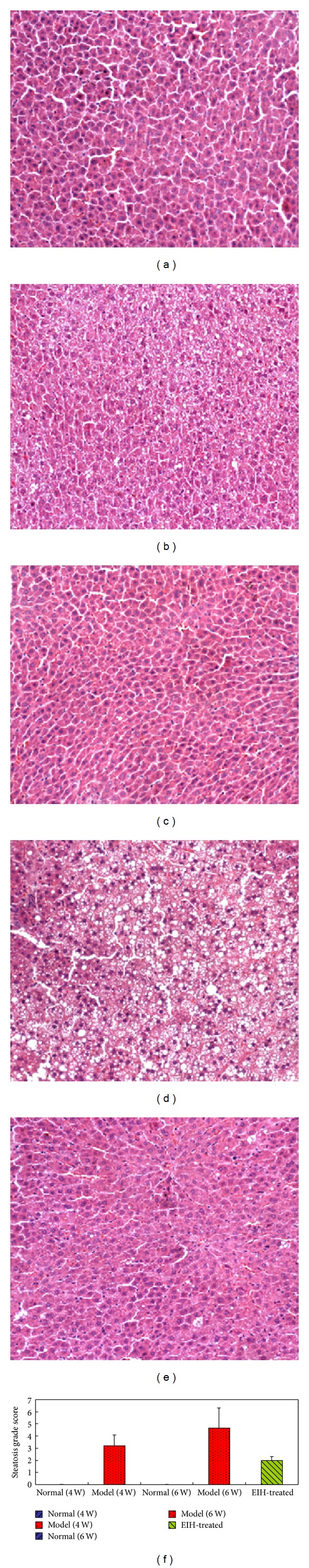
H&E staining for histological evaluation. Typical photographs of liver sections of normal rat (a) and NAFLD rat (b) in 4 weeks, normal rat (c) and NAFLD rat (d) in 6 weeks, and the rat treated with EIH (250 mg/kg) for 2 weeks (e). The steatosis grade scores of rats from each group (f). (Magnification 200x).

The feeding of high-fat diet for 4 weeks effectively induced NAFLD in rats, as evidenced by the markedly increased body weight, liver weight, liver index, and plasma TG, TC, LDL-c, ALT, and AST, while decreasing HDL-c (Table 1). Furthermore, H&E staining results (Figure 2(b)) showed that steatosis was developed and numerous lipid droplets were observed in all of the five livers from the NAFLD rats. This high-fat-diet-induced NAFLD model has the same key pathological features as those reported [2, 21] for other rat NAFLD models. Based on the previous results, the NAFLD model was considered to be successfully established. 

By contrast, administration with EIH could effectively improve these symptoms, as demonstrated by the marked decrease in body weight (by 2.01%; *P* < 0.01), liver weight (by 10.23%; *P* < 0.05), and liver index (liver/body weight, by 29.25%; *P* < 0.01) compared with the NAFLD rats (Table 1). Besides, compared with NAFLD rats, concentrations of plasma lipids, including TG, TC, and LDL-c, significantly decreased nearly one time after EIH administration, while the HDL-c content increased by 44.48% (*P* < 0.001) (Table 1). The levels of ALT and AST also showed remarkable decrease by 6.32% and 30.26%, respectively, in EIH-treated rats (Table 1). Meanwhile, hepatic histopathological examination showed the steatosis area and the number of lipid droplets were decreased in the EIH-treated group, indicating the improvement of liver steatosis (Figure 2(e)).

### 3.2. LC-Q-TOF Method Validation

System stability was evaluated by analysis of a QC sample seven times at the beginning of the batch and then after every three samples. Three common ions in positive ion mode and in negative ion mode were selected for method validation, respectively. The relative standard derivations (RSDs) of these peaks were 6.37–13.76% for peak areas and 0.03–0.95% for retention times in the positive mode (6.85–13.91% for peak areas and 0.02–0.96% for retention times in the negative mode). 

Extracts from six aliquots of a random urine sample were continuously injected to evaluate the repeatability. A stable retention time for these selected ions was observed with RSD less than 0.93% in positive mode and 0.87% in negative mode. In addition, the RSD values for peak areas were varied from 8.91% to 11.04% in positive mode and from 7.09% to 11.48% in negative mode.

All the data indicated that the established sample analysis method is highly repeatable and stable and could be used for analyzing large-scale samples in metabolomic experiments.

### 3.3. Urinary Metabolite Profile in NAFLD Rats and Identification of Biomarkers

**Figure 3:**
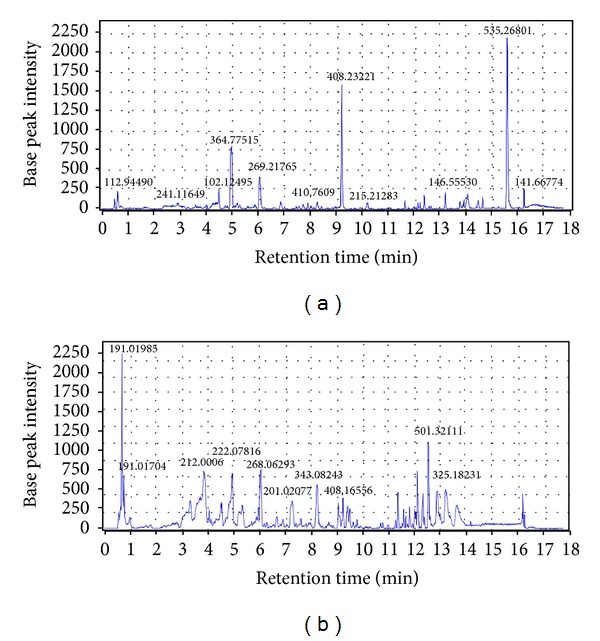
Typical urinary base peak intensity (BPI) chromatograms of normal rats in (a) ESI^+^ mode and (b) ESI^−^ mode.

Figure 3 shows typical LC-MS total ion current (TIC) chromatograms of a urine sample in positive mode and negative mode. Because a large number of signals were obtained from the urine samples, a multivariate analysis method was needed to discriminate the ions which contribute to the classification of the control and NAFLD groups.

**Figure 4:**
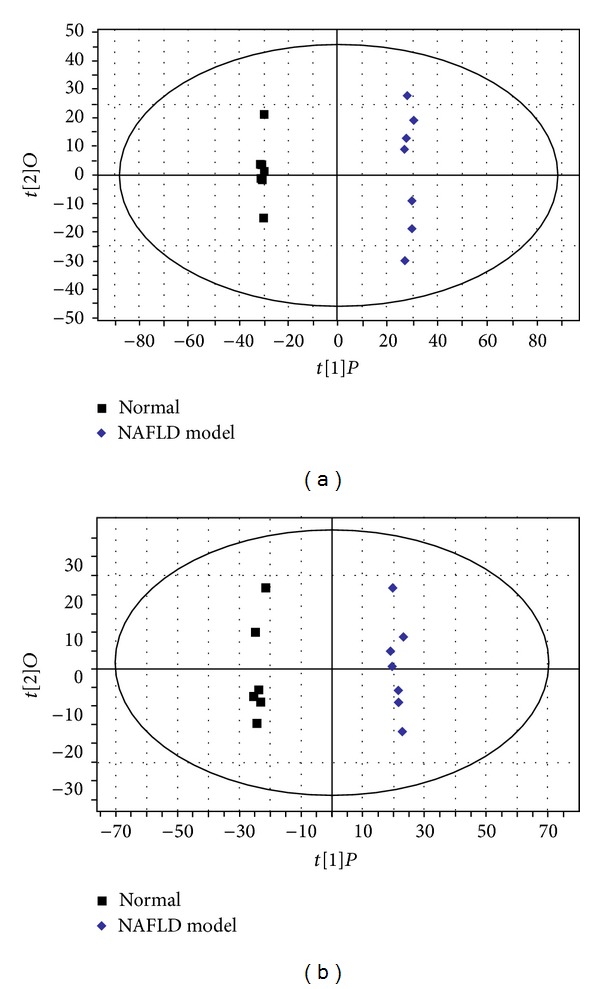
The OPLS score plots of urine samples collected from normal and NAFLD groups in (a) positive and (b) negative ion modes.

**Figure 5:**
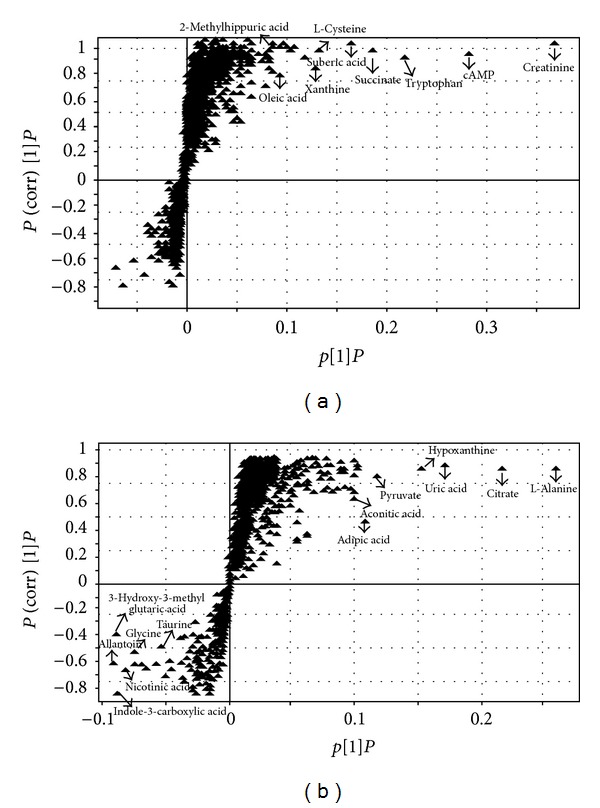
The S-plots of the OPLS model. Each triangle in the S-plot represented an ion (a) in positive and (b) in negative ion modes.

To our knowledge, orthogonal partial least squares (OPLSs) analysis is a powerful method to pick out discriminating ions that are contributing to the classification of samples and remove noncorrelated variations contained within spectra [22]. Thus, OPLS was carried out to find biomarkers of NAFLD in our study. In the OPLS score plot (Figure 4), normal and NAFLD groups were distinguished clearly, suggesting that metabolic profiles significantly changed in NAFLD model. A method validation was applied to ensure the OPLS model is reliable, and the parameters for classification from the software were *R* ^2^ *Y* = 0.999, *Q* ^2^ = 0.782 in positive mode and *R* ^2^ *Y* = 0.996,  *Q* ^2^ = 0.839 in negative mode, indicating a well-fitting OPLS-DA model had been established. To identify the role of each ion in these variations more intuitively, the S-plot was employed. The more away a triangle is from the origin, the more influence it would have on the separation of samples. Thus, the furthest metabolite ions from the origin exhibiting a higher value of variable importance projection (VIP) were potential biomarkers, which are responsible for the difference between normal group and NAFLD groups. According to the result of OPLS-DA (Figure 5), a total of 99 ions (the value of VIP > 1.0) out of 2321 variables contributed to the classification of the normal and NAFLD groups. Among these perturbed variables, 22 variables (9 in positive mode and 13 in negative mode) were predicted by comparing the accurate MS and MS/MS fragments with the metabolites searching in databases (http://metlin.scripps.edu/, http://www.hmdb.ca/), and then 7 of them were confirmed by the commercial standards. The identification results were listed in Table 2.

**Figure 6:**
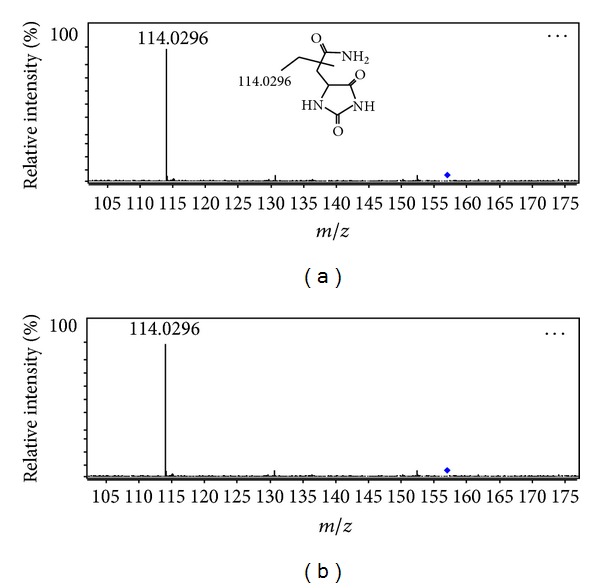
MS/MS spectra of allantoin (the precursor ion was *m/z* 157.0315; the major fragment was *m/z* 114.0296) (a) in urine samples, (b) standard.

Here, we take allantoin (*m/z* 157.0315 in negative mode) as an example to illustrate the process of biomarker identification. Its MS/MS spectra are shown in Figure 6. First the quasi-molecular ion was found out to be a mass peak at *m/z* 157.0315 (retention time was 0.656 min in negative mode). C_4_H_6_N_4_O_3_ was calculated as the most probable molecule formula, and MS/MS information was used to study its molecular structure. The above information was also searched for in Internet databases. Then, considering the elemental composition, fragmentation pattern, and chromatographic retention behavior, the *m/z* of 157.0135 was thought to be probably allantoin. This was the confirmed by comparing with commercial standard.

Among those 22 identified biomarkers, 16 were upregulated in the urine of NAFLD rat while 6 were depressed compared with normal rats (Table 2).

### 3.4. Biomarker Pathways

**Figure 7:**
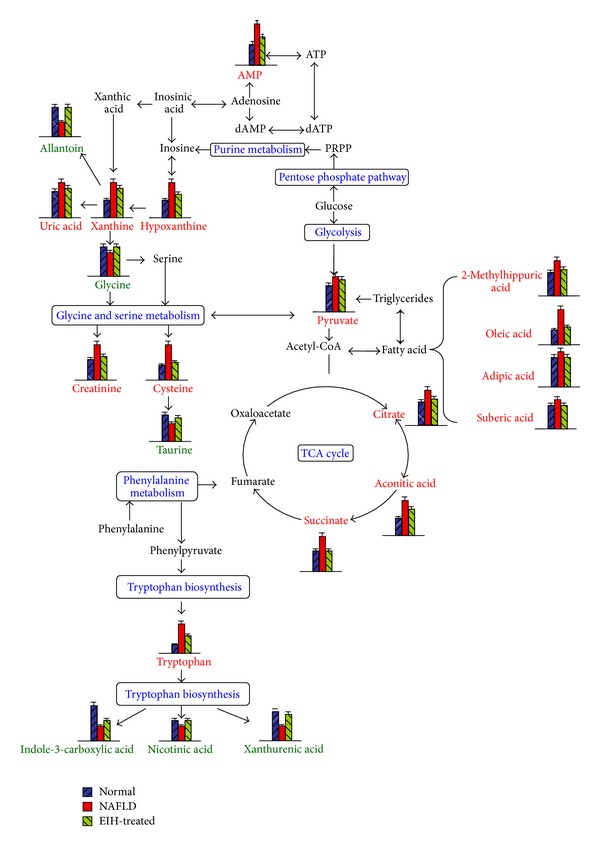
The network of the potential biomarkers changing for NAFLD and EIH-treated modulation according to the KEGG PATHWAY database. Column value in histograms is expressed as mean ± S.D. Metabolite names in red and green represent elevation and inhibition, respectively. Metabolite names in black mean they were not detected in our experiment. The bule words are pathway's names. PRPP: phosphoribosyl pyrophosphate.

In this study, 22 identified biomarkers were identified by LC-Q-TOF. The related pathway of each biomarker was also recorded in Table 2 by searching the KEGG PATHWAY Database (http://www.genome.jp/kegg/). By relating the metabolic pathways, the metabolic network of the potential biomarkers was established and shown in Figure 7.

As shown in Figure 7, we found that most pathways of the potential biomarkers were related to energy metabolism, including the citrate cycle, purine metabolism, amino acid metabolism, tryptophan metabolism, and fatty acid metabolism. Among which, the citrate cycle is the central biological process in this network. In this study, the citrate cycle was activated in NAFLD rats, as evidenced by the increased level of citrate cycle intermediates, including pyruvate, aconitic acid, citrate, and succinate. This result is compatible with the results of previous studies [23]. It has been reported that pyruvate in the urine samples was elevated in a high-fat-diet-fed group due to the inhibition of pyruvate dehydrogenase [24]. NAFLD is characterized by fat deposits in liver, which may be induced by high-fat diet [2]. In agreement with this, we can observe the elevation of free fatty acids (FFA) such as suberic acid, oleic acid, and adipic acid in the urine. High concentrations of fatty acids have been used as an indicator of disease risk [23]. Meanwhile, the increased provision of FFA causes an increase in FFA oxidation, resulting in increasing the concentration of citrate cycle intermediates, including aconitic acid, citrate, and succinate.

In this study, five biomarkers were related to purine metabolism. The levels of cAMP, hypoxanthine, xanthine, and uric acid were elevated, indicating the purine metabolism was enhanced. One possible explanation for this might be DNA damage and apoptotic cell death were caused by the increased reactive oxygen species resulting from excess dietary fat under the condition of NAFLD [25]. Hypoxanthine could be metabolized to xanthine and uric acid by xanthine dehydrogenase or xanthine oxidase [22]. It has been considered that xanthine oxidase generated excess oxygen-free radicals and then caused liver injury in pathways [22]. Meanwhile, cAMP is a metabolite of ATP [18]. The reason for its accumulation in urine of NAFLD rats is probably related to the degradation of ATP. Uric acid is the final oxidation product of purine metabolism. The increased of uric acid level is not only an epiphenomenon of metabolic alterations, but also a factor directly involved in the pathogenesis of diseases. It has been reported that allantoin was a marker of oxidative stress [22]. Generally, the level of allantoin should increase in NAFLD model. However, it decreased in our experiments. Although the reason is not clear, this result is similar to [22].

Endogenous creatinine is a breakdown product of creatine in muscle, which is biosynthesized from arginine and glycine [26]. In vivo, creatinine is usually produced at a fairly constant rate proportional to muscle mass and then filtered from the blood by the kidneys [27]. In NAFLD model, skeletal and cardiac muscle might be hypertrophy to support and move the increased body mass [28]. Meanwhile, endogenous biosynthesis pathways and kidney function might be altered in NAFLD model [29]. Therefore, all of these might contribute to the increased urinary excretion of creatinine in NAFLD model. Similar results were observed in previous studies [28]. In contrast, due to the increased degradation of creatine, its biosynthesis from biomolecules, such as glycine, was compensatorily elevated in response to NAFLD.

In addition, taurine has many important biological roles such as conjugation of cholesterol and bile acids, antioxidation, osmoregulation, and modulation of calcium signaling [30]. It has been reported that taurine as a metabolite could increase fatty acid oxidation and decrease obesity [31]. In light of this, as high-fat diet could provide more fatty acids, it would require more taurine to promote fatty acid oxidation. Thus, the excretion of taurine in urine was decreased.

The metabolite profiles also showed the increase of L-tryptophan in NAFLD model. One possible explanation was that the liver injury leads to its metabolic remodeling. Meanwhile, nicotinic acid, xanthurenic acid, Indole-3-carboxylic acid, and metabolites of L-tryptophan decreased in NAFLD model, which may further predict the abnormality in the L-tryptophan metabolism in NAFLD model.

In summary, these 22 potential biomarkers mainly associated with citrate cycle, purine metabolism, amino acid metabolism, tryptophan metabolism, and fatty acid metabolism provided a new insight into the development and progression of NAFLD.

### 3.5. Effects of EIH on NAFLD Based on Metabolite Profile

The therapeutic effect of EIH on the treatment of NAFLD can be demonstrated not only from Section 3.1 but also in metabolomic study. As the 22 potential biomarkers for NAFLD have been found, we further took them as the monitoring indexes for investigating the therapeutic effects of EIH by PCA and OPLS-DA. 

**Figure 8:**
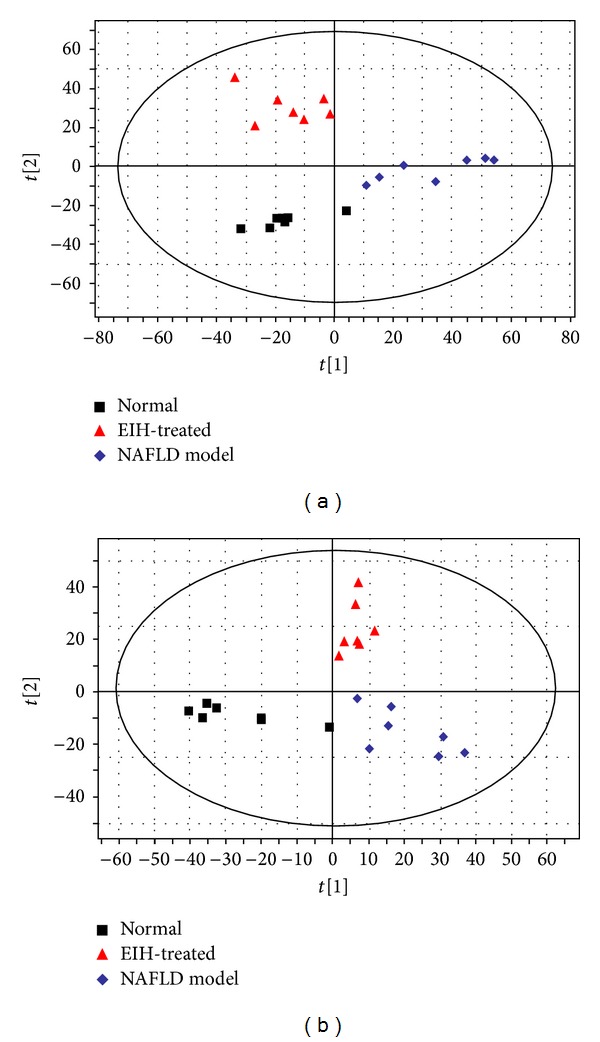
The PCA score plots of urine samples collected from normal, NAFLD model, and EIH-treated groups in (a) positive and (b) negative ion modes.

Initially, PCA was applied. The *R* ^2^ *X* and *Q* ^2^ were 0.48 and 0.213 in positive mode and 0.57 and 0.286 in negative mode, which indicated the classifications were well for PCA models. The score plots (Figure 8) of the first two principal components allowed visualization of the data and comparing of the three group. The normal, NAFLD, and EIH treatment groups are classified clearly.

**Figure 9:**
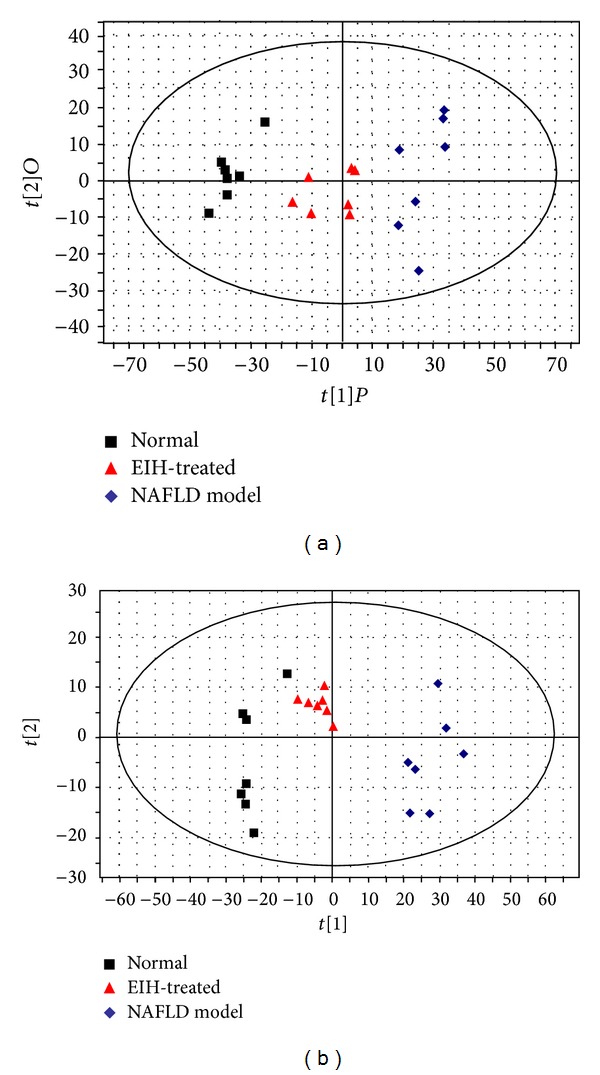
The OPLS score plots of urine samples collected from normal, NAFLD model, and EIH-treated groups in (a) positive and (b) negative ion modes.

Following data analysis using PCA, the data was further examined using OPLS-DA. The established OPLS-DA model was reliable according to the validation (*R* ^2^ *Y* = 0.943, *Q* ^2^ = 0.702 in positive mode and *R* ^2^ *Y* = 0.947, *Q* ^2^ = 0.732 in negative mode). The score plot of the OPLS-DA model (Figure 9) showed that normal, NAFLD, and EIH treatment groups are classified better than PCA, and the EIH treatment group is closer to the normal group than the NAFLD group, which might suggest that EIH can reverse the pathological process of NAFLD.

**Figure 10:**
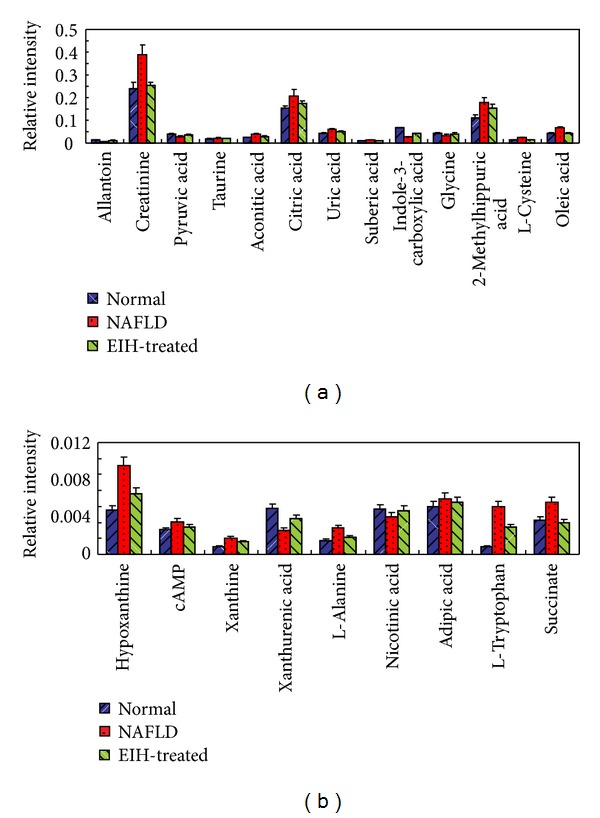
Bar plots show UPLC-MS relative signal intensities for 22 metabolites ((a) and (b)) in normal, NAFLD, and EIH-treated groups. Data are expressed as mean ± S.D.

To further evaluate the reversed condition of the potential biomarkers by administration of EIH, Student's *t*-test was performed and the value of *P* was set to 0.05 for significantly differential variables in this study. The relative peak intensities of the 22 metabolites to their respective total integrated are shown in Figure 10. Compared to the NAFLD group, 19 metabolites were significantly reversed in EIH-treated group, and the other three metabolites were also reversed at different degrees. These results implied that EIH might functionally intervene in citrate cycle, purine metabolism, tryptphan metabolism, and amino acids metabolism. Combined with the pharmacological assay, it is demonstrated that EIH had extensive effects in the treatment of NAFLD through partially regulating the disturbed pathways of energy metabolism.

## 4. Conclusion

In this study, a metabolomic approach based on UPLC-Q-TOF-MS detection has been successfully established for biomarker exploration in NAFLD and mechanism studies of EIH. As a result, 22 metabolites were screened out and considered as potential biomarkers of NAFLD. Taking these biomarkers as possible drug targets, it is revealed that EIH could reverse the pathological process of NAFLD through regulating the disturbed pathways of metabolism including citrate cycle, purine metabolism, amino acid metabolism, tryptophan metabolism, and fatty acid metabolism. The metabolomic results not only supply a systematic view of the development and progression of NAFLD but also provide a theoretical basis for the prevention or treatment of NAFLD.

## Figures and Tables

**Table 1:** Effects of TTE on blood biochemical characteristics.

Parameter	4 w		6 w		
	Normal group	NAFLD group	Normal group	NAFLD group	EIH-treated group
Body weight (g)	279.43 ± 13.69	299.33 ± 18.94*	309.60 ± 9.79	332.60 ± 25.27^△△^	339.30 ± 21.74
Liver weight (g)	8.56 ± 0.53	12.78 ± 0.36**	8.318 ± 0.664	14.665 ± 1.98^△△^	13.165 ± 1.201^#^
Liver/body weight (%)	2.89 ± 0.21	4.53 ± 0.21**	2.87 ± 0.28	4.82 ± 0.20^△△^	3.41 ± 0.21^#^
TG (mg/dL)	53.53 ± 8.58	182.45 ± 57.65**	59.53 ± 9.022	85.75 ± 23.12^△^	52.13 ± 12.68^#^
TC (mg/dL)	58.46 ± 9.44	105.89 ± 12.69**	53.182 ± 4.506	92.65 ± 11.47^△△^	55.755 ± 8.12^##^
LDL-c (mg/dL)	7.430 ± 3.75	20.06 ± 11.67**	7.181 ± 2.52	25.63 ± 5.767^△△^	13.57 ± 7.49^#^
HDL-c (mg/dL)	42.71 ± 7.16	31.210 ± 7.27**	42.305 ± 11.10	21.31 ± 2.36^△△^	30.79 ± 6.16^#^
ALT (U/L)	3.57 ± 1.14	5.20 ± 2.10*	4.65 ± 0.133	7.12 ± 1.024^△△^	6.67 ± 0.82^#^
AST (U/L)	2.85 ± 1.25	6.78 ± 2.66**	3.723 ± 0.231	5.459 ± 1.026^△△^	3.807 ± 0.45^##^
FBG (mmol/L)	9.66 ± 1.25	9.07 ± 2.76	8.677 ± 0.219	10.38 ± 2.24	8.874 ± 0.93

*P < 0.05, **P < 0.01, compared to normal group (4 w). ^△^ *P* < 0.05, ^△△^ *P* < 0.01, compared to normal group (6 w); ^#^ *P* < 0.05, ^##^ *P* < 0.01, compared to NAFLD group (6 w).

**Table 2:** Identification of significantly differential metabolites in the rat urine and their related pathways.

Metabolite	RT	Mass	Ion (*m*/*z*)	Formula	Ion mode	Model trend^a^	Treatment trend^b^	Related pathway
Allantoin	0.656	158.0393	157.0315	C_4_H_6_N_4_O_3_	ESI^−^	(∗)↓	(∗)↑	Purine metabolism
Hypoxanthine	0.672	136.0379	135.0301	C_5_H_4_N_4_O	ESI^−^	(∗)↑	(∗)↓	Purine metabolism
Creatinine	0.679	113.0563	114.0641	C_4_H_7_N_3_O	ESI^+^	(∗)↑	(∗)↓	Arginine and proline metabolism
Taurine	0.694	125.0150	124.0072	C_2_H_7_NO_3_S	ESI^−^	(∗)↓	(∗)↑	Taurine and hypotaurine metabolism
Pyruvate	0.716	88.0138	87.0060	C_3_H_4_O_3_	ESI^−^	(∗)↑	(∗)↓	Alanine metabolism; citric acid cycle; cysteine metabolism Glycolysis/gluconeogenesis; glycine and serine metabolism
Aconitic acid	0.735	174.0213	173.0135	C_6_H_6_O_6_	ESI^−^	(∗)↑	(#)↓	Citrate cycle
Citrate	0.737	192.0252	191.0174	C_6_H_8_O_7_	ESI^−^	(∗)↑	(∗)↓	Citric acid cycle
Uric acid	0.775	168.0264	167.0186	C_5_H_4_N_4_O_3_	ESI^−^	(∗)↑	(∗)↓	Purine metabolism
Suberic acid	0.814	174.0846	175.0924	C_8_H_14_O_4_	ESI^+^	(∗)↑	(∗)↓	Oleic acid degradation
cAMP	1.885	329.0545	330.0623	C_10_H_12_N_5_O_6_P	ESI^+^	(∗)↑	(∗)↓	Purine metabolism
Xanthine	2.427	152.0309	153.0387	C_5_H_4_N_4_O_2_	ESI^+^	(∗)↑	(∗)↓	Purine metabolism
L-Alanine	2.807	89.0475	88.0397	C_3_H_7_NO_2_	ESI^−^	(∗)↑	(∗)↓	Alanine metabolism; glucose-alanine cycle; glycine and serine metabolism
Nicotinic acid	3.802	123.0314	122.1094	C_6_H_5_NO_2_	ESI^−^	(∗)↓	(∗)↑	Tryptophan metabolism; nicotinate and nicotinamide metabolism
Adipic acid	4.081	146.0562	145.0484	C_6_H_10_O_4_	ESI^−^	(∗)↑	(∗)↓	Fatty acid metabolism
Indole-3-carboxylic acid	4.535	161.0475	160.0397	C_9_H_7_NO_2_	ESI^−^	(∗)↓	(∗)↑	Tryptophan metabolism
3-Hydroxy-3-methyl-Glutaric acid	4.563	162.0512	161.434	C_6_H_10_O_5_	ESI^−^	(∗)↓	(∗)↑	A metabolite that accumulates in the urine of patients affected by 3-hydroxy-3-methylglutaric aciduria caused by reduced enzyme activity of the intramitochondrial 3-Hydroxy-3-methylglutaryl-CoA lyase, which catalyzes the final step of leucine degradation and plays a key role in ketone body formation.
Glycine	5.225	75.0320	74.0242	C_2_H_5_NO_2_	ESI^−^	(∗)↓	(∗)↑	Glycine, serine, and threonine metabolism
2-Methylhippuric acid	5.244	193.0725	194.0803	C_10_H_11_NO_3_	ESI^+^	(∗)↑	(∗)↓	Fatty acid metabolism
L-Tryptophan	5.830	204.0929	205.1007	C_11_H_12_N_2_O_2_	ESI^+^	(∗)↑	(∗)↓	Tryptophan metabolism
Succinate	5.974	118.0267	119.0346	C_4_H_6_O_4_	ESI^+^	(∗)↑	(∗)↓	Citric acid cycle;
L-Cysteine	6.250	121.0191	122.0269	C_3_H_7_NO_2_S	ESI^+^	(∗)↓	(#)↓	Glycine, serine, and threonine metabolism;
Oleic acid	14.133	282.2626	283.2704	C_18_H_34_O_2_	ESI^+^	(∗)↓	(#)↓	Fatty acid biosynthesis

^a^Compared to normal group. ^b^Compared to model group. Arrow (↑) indicates relative increase in signal. Arrow (↓) indicates relative decrease in signal. Asterisk (∗) represents a statistically significant difference (*P* < 0.05), while pound key (#) represents no statistically significant difference.
